# The effects of combined environmental factors on the intestinal flora of mice based on ground simulation experiments

**DOI:** 10.1038/s41598-021-91077-7

**Published:** 2021-05-31

**Authors:** Peiming Sun, Jiaqi Yang, Bo Wang, Huan Ma, Yin Zhang, Jinhu Guo, Xiaoping Chen, Jianwei Zhao, Hongwei Sun, Jianwu Yang, Heming Yang, Yan Cui

**Affiliations:** 1 Department of General Surgery, Strategic Support Force Medical Center, Chaoyang District, Beijing, 100101 China; 2 Department of General Surgery, The 306th Hospital of Chinese PLA-Peking University Teaching Hospital, Chaoyang District, Beijing, 100101 China; 3 China Astronaut Research and Training Center, Haidian District, Beijing, 100094 China; 4 Ministry of Education (MOE) Key Laboratory of Gene Function and Regulation, State Key Laboratory of Biocontrol, School of Life Sciences, Sun Yat-Sen University, Haizhu District, Guangzhou, 510006 China

**Keywords:** Microbiology, Bacteria, Environmental microbiology, Fungi

## Abstract

The composition and function of intestinal microbial communities are important for human health. However, these intestinal floras are sensitive to changes in the environment. Adverse changes to intestinal flora can affect the health of astronauts, resulting in difficulties in implementing space missions. We randomly divided mice into three groups and placed each group in either a normal environment, simulated microgravity environment or a combined effects environment, which included simulated microgravity, low pressure and noise. Fecal samples of the mice were collected for follow-up analysis based on metagenomics technology. With the influence of different space environmental factors, the species composition at the phylum and genus levels were significantly affected by the combined effects environment, especially the abundance of the *Firmicutes* and *Bacteroidetes*. Furthermore, screening was conducted to identify biomarkers that could be regarded as environmental markers. And there have also been some noticeable changes in the function of intestinal floras. Moreover, the abundance of antibiotic resistance genes (ARGs) was also found to be changed under different environmental conditions, such as bacitracin and vancomycin. The combined effects environment could significantly affect the species composition, function, and the expression of ARGs of intestinal flora of mice which may provide a theoretical basis for space medical supervision and healthcare.

## Introduction

The intestines of the human body harbor and reside a diverse population of symbiotic bacteria. Under normal circumstances, the composition of intestinal flora is relatively stable and maintains a state of harmonious symbiosis with the body^1^. More importantly, the microbiota plays a pleiotropic role in human health and disease as a gigantic community in the intestines^2^. It is involved in the digestion and absorption of nutrients from food on the one side^3^. For example, gut microbiota can regulate the metabolism of short-chain fatty acids (SCFAs)^4–6^, vitamins^7^ and amino acids^8^. On the other hand, engaging with humans to induce and educate the immune system for pathogen defense and contribute to the maturation of the intestinal epithelium, has a significant impact on immune system development and homeostasis^9–11^. Specifically, several studies have proven that the development and improvement of the human immune system cannot be achieved without intestinal floral diversity^12–14^. Slow development and weakened immune systems have been found in rodent models with sterile intestines^15^. When the immune system of the body is weakened, opportunistic infections caused by opportunistic pathogens, such as fungi or *staphylococci*.

The outer space environment is an extremely dangerous environment filled with more threats than the terrestrial environment^16^. Common variation factors include weightlessness, intense radiation, extreme cold, and hypoxia^17^. Furthermore, astronauts on the International Space Station (ISS) spend the majority of their time inside the spacecraft, where they are exposed to a noisy atmosphere. The fans, pumps, and ventilation associated with environmental protection, life support, and thermal control systems are the primary sources of noise^18^. Crew operation has been blamed for other noise limit violations^19^. Existing studies have confirmed that the human body experiences certain physiological and pathological changes in space environments, which are mainly manifested as reduced bone density^20,21^, reduced orthostatic tolerance^22^, abnormal digestive function^23–26^, and immune dysfunction^27^. Moreover, the composition and activity of gut microbes may be changed after they were exposed to endogenous and environmental factors^28^, as well as the intestinal microecology of the organism, which may undergo adverse changes in space environments. Meanwhile, the pathogenicity and resistance of intestinal flora may also be enhanced in space environment, resulting in disruption of the balance between organisms and flora^29–31^. These changes in immune function and intestinal microbiota may increase the risk of developing diseases. Previous spaceflight studies have shown that astronauts were easily infected by bacteria, viruses, or opportunistic pathogens^30,32,33^. In addition, if the astronaut has a history of using large doses of antibiotics due to chronic intestinal diseases, treatment for infections would also be extremely difficult^34^.

The space environment is full of complex environmental changes, and most existing ground-based simulation studies have explored the effects of single variables on organisms, such as focusing on microgravity^25^, low pressure^35^, or noise^36,37^. Moreover, functional changes of the astronaut may result in interactions between various environmental factors. Therefore, we designed this experiment to explore changes in the intestinal flora of mice affected by the combined effects environment, which included microgravity, low pressure, and noise environments, to provide the changes of microbiota for medical supervision and medical insurance for astronauts of the immune or digestive diseases.

**Figure 1:**
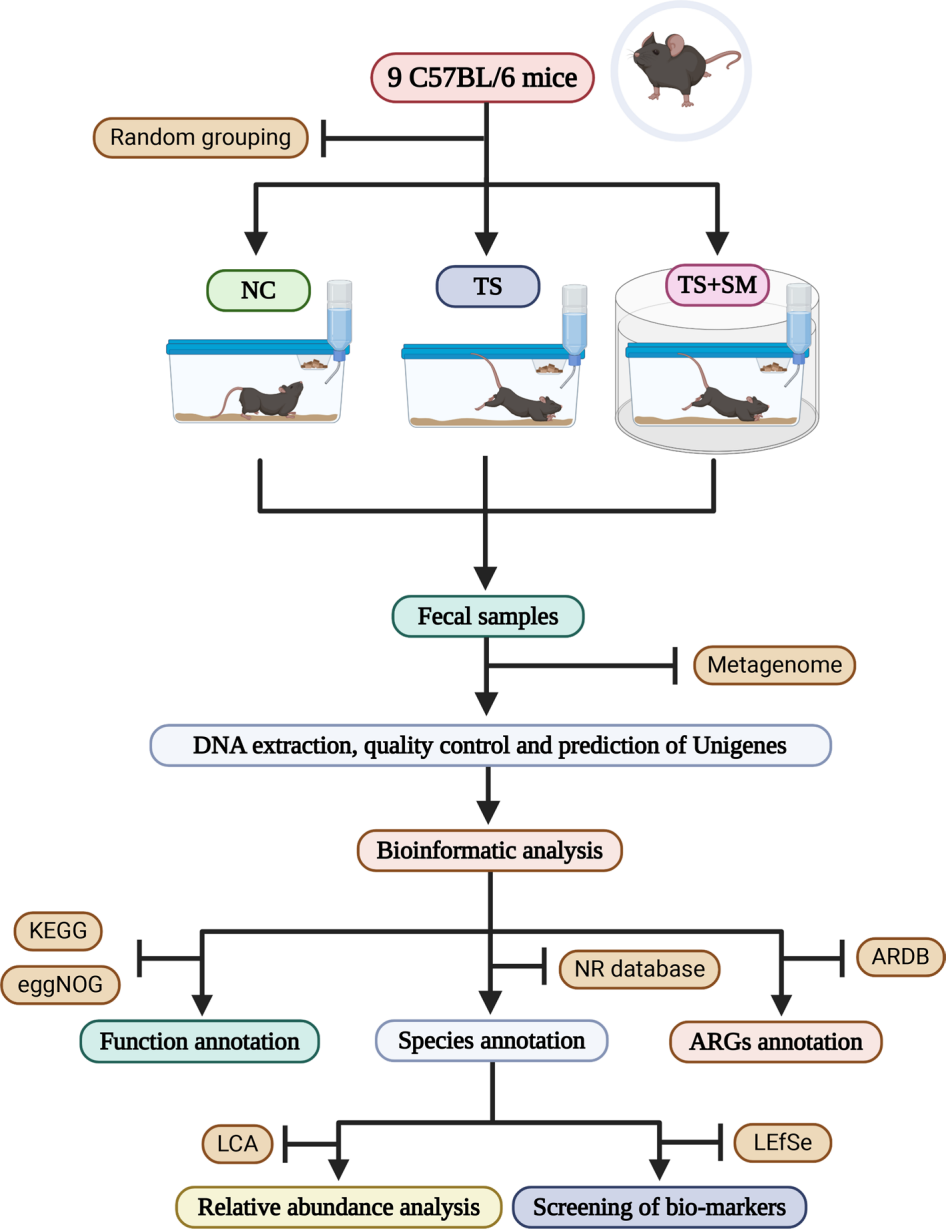
The workflow diagram of the present study.

The intestinal microorganism community constitutes a gene pool that is large and complex and contains both phylogenetic marker genes, as well as various metabolism genes, which are collectively known as the metagenome^38^. Researchers can analyze and forecast these genes to study the specific composition and function of the microbial community. For example, the phylogenetic markers of different microbial identities of 16S rRNA genes could be used to perform species identification. In this study, fecal samples of mice in the three different groups were collected and a large quantity of biological data and an abundance of information on microorganisms were obtained using Next-Generation Sequencing. Then, a bioinformatics analysis of these data was performed to further elucidate the composition and functional changes and the abundance of resistance genes of the gut microbes in the intestine. The project workflow is shown in Fig. 1 which was drawing with the BioRender's material (https://app.biorender.com/). Furthermore, we expect that the results of this study could provide a theoretical basis for the maintenance of normal intestinal microecology of astronauts.

## Results

### Screening and identification of differentially expressed genes

**Figure 2:**
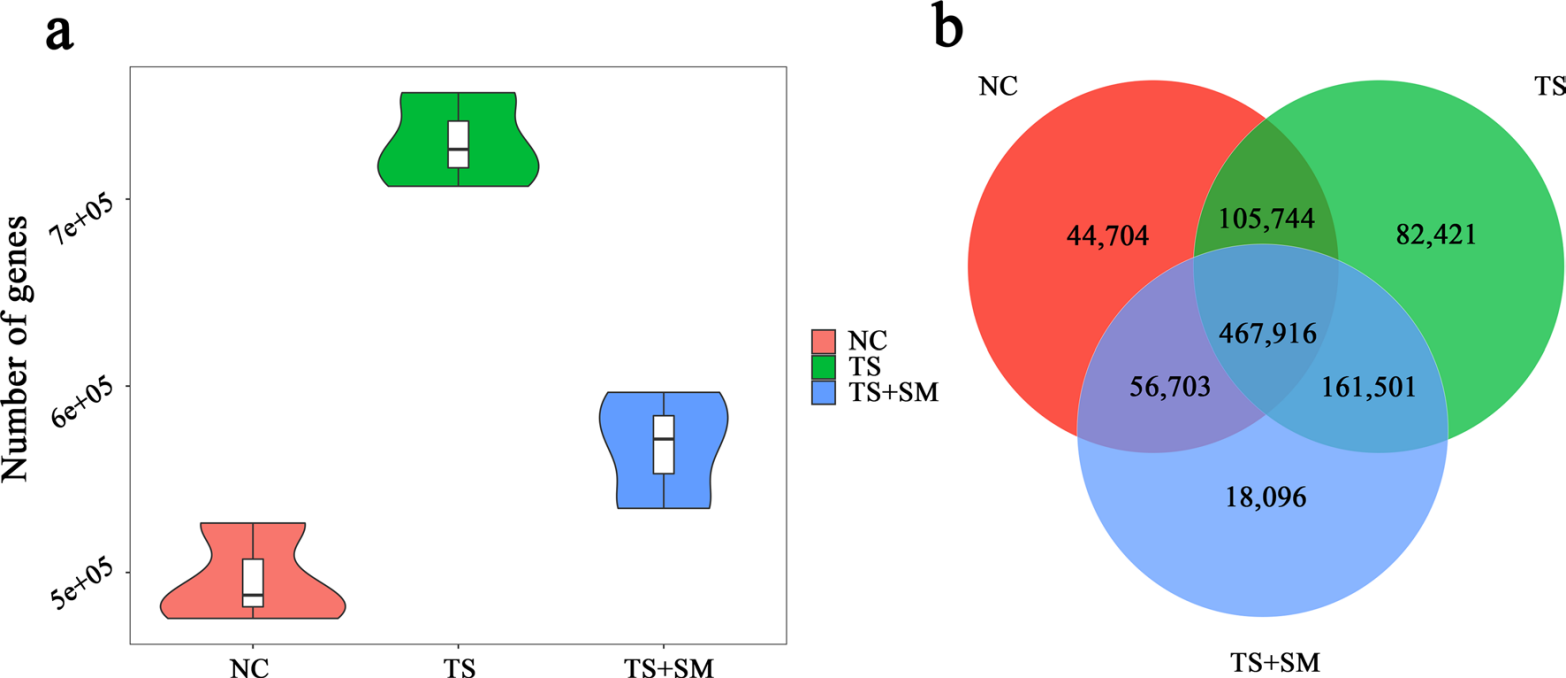
The results of the prediction of genes and their relationships. (**a**) The number of genes in the three groups is shown in the violin plot. (**b**) The intersection of the predicted genes among the three groups.

We obtained a large amount of raw data (Table S1) through the extraction and sequencing of DNA from mice stool samples. After further assembling and screening of raw data, scaftigs were obtained to be used for gene prediction (Table S2). The results of the prediction and comparison between the Open Reading Frames (ORFs) are shown in Table S3. A total of 939,480 effective genes were obtained through the above-mentioned process. It was observed that there were significant differences in the number of effective genes among the three groups (Fig. 2a). Further analysis showed that the total number of common genes between the three groups was 467,916, while 44,704, 82,421, and 18,096 genes were differentially expressed between the three groups (Fig. 2b).

### Combined effects environment significantly affected the species composition of gut microbiota in mice

#### Overall results of species annotation

**Figure 3:**
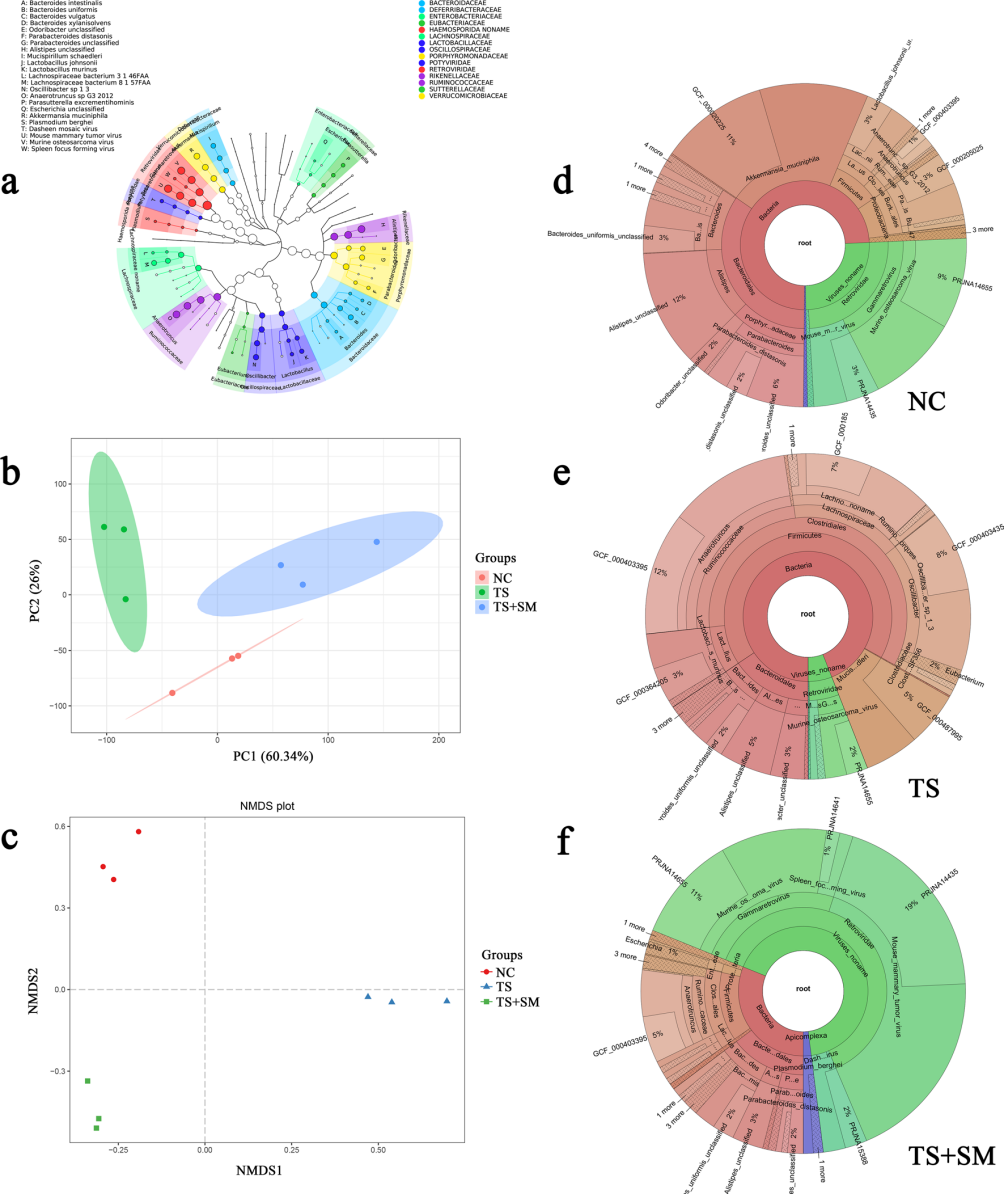
The overall results of species annotation and the correlation analysis of all samples. (**a**) Merged species information of all three groups based on GraPhlAn. (**b**,**c**) The PCA and NMDS analysis of all samples. (**d**–**f**) d, e, and f represent the species annotation of the NC, TS, and TS + SM groups, respectively.

We used metaphlan2 software to predict effective genes and analyze species-related information. The merged information obtained from all species was visualized to determine the taxonomic composition of the intestinal flora of the mice based on GraPhlAn (Fig. 3a). From the inside out, the diagram depicts annotated species in phylum to genus order. The average relative abundance of species is represented by node size, and the same shade color corresponds to the same species source. To further verify the differences in species composition among samples from different groups, principal component analysis (PCA) and non-metric multidimensional scaling (NMDS) analysis were introduced to analyze the data. The horizontal axis of the PCA (Fig. 3b) represents sample scores for the first principal components, while the vertical axis represents sample scores for the second principal components. 60.34% of variances from 9 samples were explained by the first principal components, while 26% of variances from 9 samples were explained by the second principal components. The total degree of variance resolution was 86.34%. The distance between samples of the same group is relatively close in the NMDS (Fig. 3c), whereas the distance between samples of different groups is relatively far. This indicates that there are significant differences in species classification among the groups in our samples. These results can be further presented in the species annotation obtained using KRONA software based on the mean value of three groups is shown in Fig. 3d–f. The different taxonomic levels from inside to outside are shown by the circles in the diagram, and the fan size represents the relative proportion of species.

#### Relative abundance and cluster analysis of species

**Figure 4:**
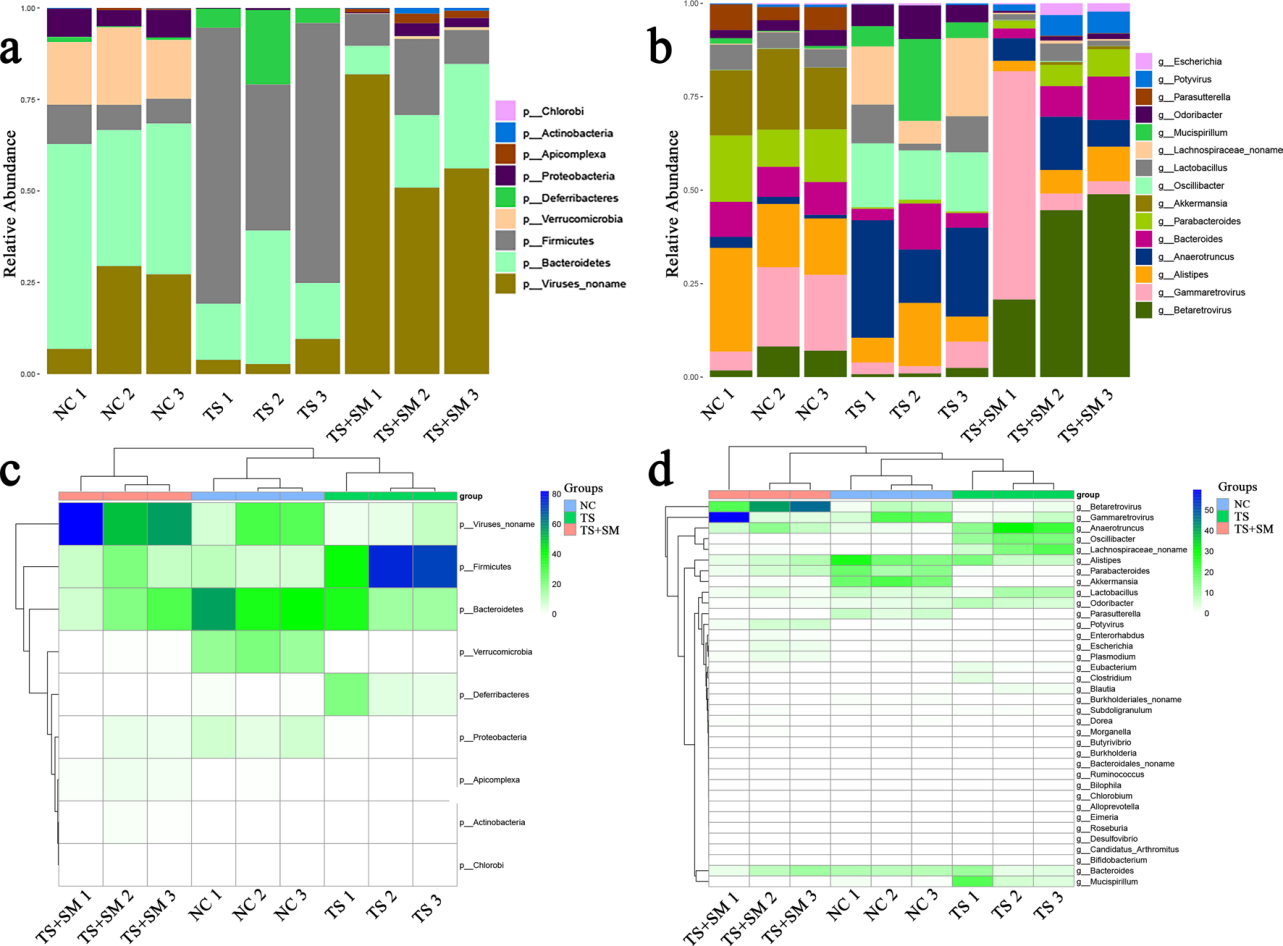
The relative abundances of species and heat maps of all samples. (**a**,**b**) The relative species abundance at phylum level and genus level. (**c**,**d**) The heat map of species abundance at phylum level and genus level.

To further clarify the differences of species between different groups, we analyzed the relative abundance of species within different groups. The relative abundances histogram of the top 15 species in each sample were drawn using the corresponding species annotation results based on the relative abundance of different classification levels (Fig. 4a,b). The species with the highest relative abundance ranking top 36 in each sample were used to construct the cluster treemap at phylum and genus levels (Fig. 4c,d). At the phylum level, the changes of relative abundances of *Firmicutes*, *Bacteroides,* and *Verminobacteria* are worth paying attention to. The abundance of *Firmicutes in* the TS group was increased in comparison to the NC and TS + SM groups (NC vs. TS *P* = 0.0087; TS + SM vs. TS *P* = 0.0142). However, there was no change between the NC group and the TS + SM group (TS + SM vs. NC *P* = 0.3127). Compared with the NC group, the relative abundance of *Bacteroides* only showed a downward trend in the TS + SM group (TS + SM vs. NC *P* = 0.0352; TS vs. NC *P* = 0.0692). Meanwhile, *Verrucomicrobia* almost disappeared from the TS + SM group (TS + SM vs. NC *P* = 0.0004). At the genus level, the relative abundance of *β-retroviruses* showed a significant increase in the TS + SM group (NC vs. TS + SM *P* = 0.0215), compared with the NC group. The abundance of *Lachnospiraceae* in the TS group was increased in comparison to the NC and TS + SM groups (NC vs. TS *P* = 0.035; TS + SM vs. TS *P* = 0.0377). However, there was no change between the NC group and the TS + SM group (TS + SM vs. NC *P* = 0.06). The relative abundance of *Anaerotruncus* and *Oscillibacter*, in particular, differed significantly and followed a similar pattern. The TS group and TS + SM group showed a large increase in comparison to the NC group (NC vs. TS *P* = 0.0151, 0.0003, respectively; NC vs. TS + SM *P* = 0.0452, 0.0003, respectively), while the abundance between the TS group and the TS + SM group experienced no improvement (TS + SM vs. TS *P* = 0.06). Moreover, Akkermansia almost disappeared from the TS + SM group (NC vs. TS + SM *P* = 0.0004).

#### Screening of representative biomarkers

**Figure 5:**
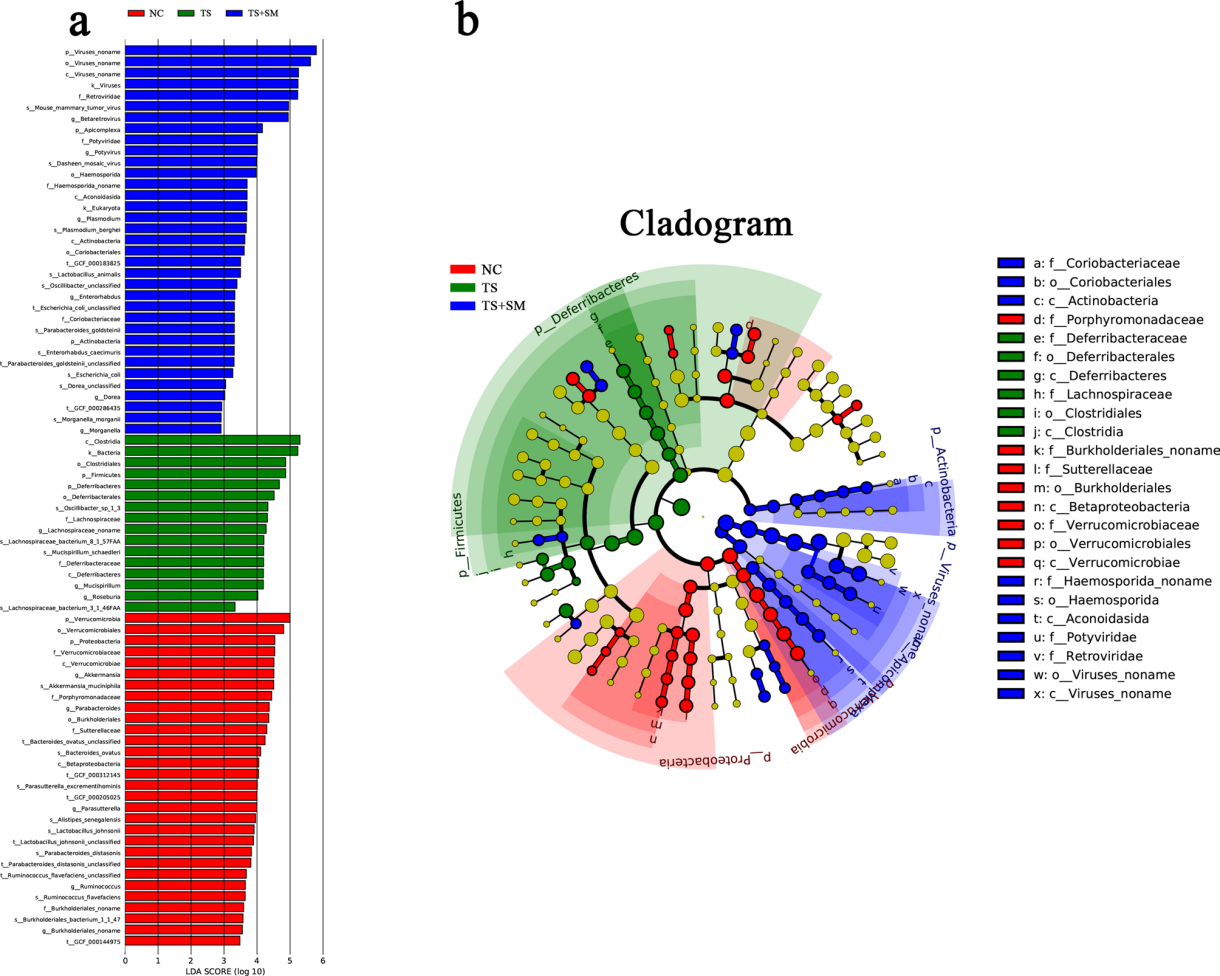
The results of the screening of biomarkers from all three groups. (**a**) The LEfSe score of the biomarkers in the three groups. (**b**) The cladogram of representative biomarkers and their evolutionary relationship. *Note* The Biomarkers with no significant difference were uniformly colored yellow. The different color nodes represented the microbial groups that played an important role in the three groups.

To explore the differences among species in the gut microbiota that were affected by weightlessness or combined effect environment, we carried out a Linear discriminant analysis of Effect Size (LEfSe) analysis to obtain species-specific biomarkers. The gut microbial compositions of three groups were compared to obtain the Linear discriminant analysis (LDA) score of each species. Biomarkers with statistical significance were listed in Fig. 5a (only biomarkers with score ≥ 3 were shown, the length of the bar chart represents the impact of different species). After exposure to the environments with weightlessness or combined effect environment, the abundances of 28 species decreased in the NC group, while the abundances of 24 and 32 species decreased at all levels in the TS group and TS + SM group, respectively. *f-Bacteroidales*, *f-Deferribacteraceae*, and *f-Coriobacteriaceae* were found to be the biomarkers with the highest scores in the NC group, TS group, and TS + SM group, respectively. All biomarkers with a significant differences are shown in the cladogram of species differences at all levels for each different species (Fig. 5b).

### Combined effects environment significantly affected the function of gut microbiota of mice

**Figure 6:**
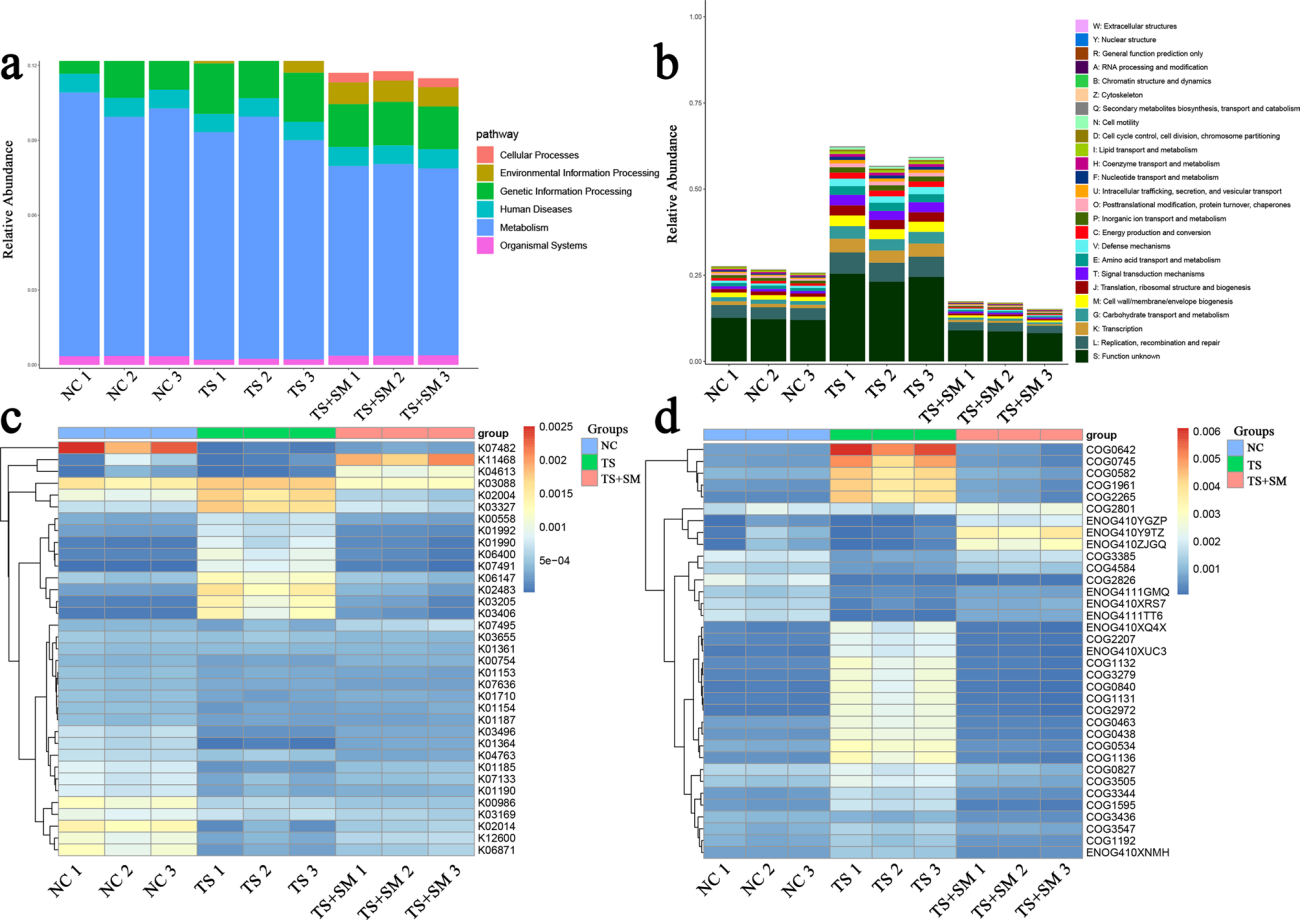
Functional enrichment analysis of all samples. (**a**,**b**) The results of the functional enrichment analysis of all samples using the KEGG and eggNOG databases. (**c**,**d**) The heat map showing the enriched functions of all samples obtained using the KEGG and eggNOG databases.

To investigate the influence of different environments on gut microbiota function, we blasted the Unigenes to the Kyoto Encyclopedia of Genes and Genomes (KEGG) and evolutionary genealogy of genes: Non-supervised Orthologous Groups (eggNOG) databases. The results showed that the microgravity or combined environments markedly affected the gut microbiota function. In the KEGG database (Fig. 6a), compared with the NC group, the proportion of metabolism in the TS and TS + SM groups decreased, while the decrease was most obvious in the TS + SM group. The pathways of Environmental Information Processing and Cellular Processes of the TS + SM group and TS group increased. Based on the eggNOG database (Fig. 6b), the relative proportion of various functions in the TS + SM group decreased significantly while the same increased in the TS group, compared with the NC group. However, certain changes deserve further attention. Specifically, the function involving replication, recombination, and repair, and transcription increased in the TS group but decreased significantly in the TS + SM group. All functional changes detected by the two databases were clustered and were shown in Fig. 6c,d.

### Identification and classification of ARGs

**Figure 7:**
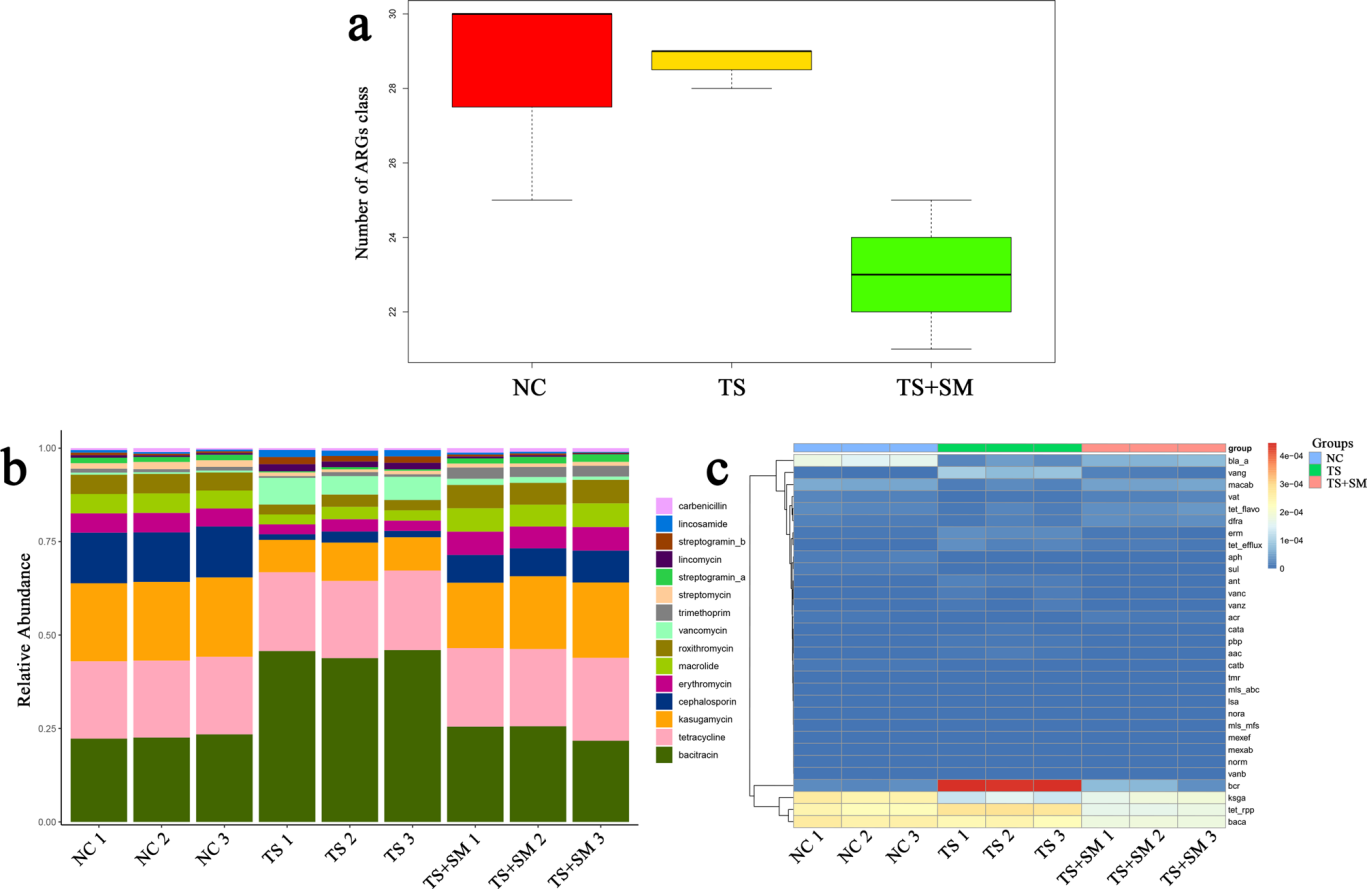
The predicted ARGs and their relative abundance in all samples. (**a**) The number of ARGs identified in the three groups. (**b**) The relative abundances of the top 15 ARGs in all samples. (**c**) The cluster heat map of the ARGs in all samples.

We used the Antibiotic Resistance Genes Database (ARGD) to annotate antibiotic resistance genes. Based on the results of the comparison between the Unigenes and the data in the ARGD database, we performed differential analyses of the ARGs in all three groups (Fig. 7a). Then, the ARGs were used to predict resisted antibiotics and the relative abundance of the histogram of the top 15 ranking antibiotics was drawn. As shown in Fig. 7b, compared with the NC group, the abundance of bacitracin and vancomycin increased in the TS group (bacitracin, NC vs. TS *P* = 0.000079483; vancomycin, NC vs. TS *P* = 0.0006), while kasugamycin decreased (NC vs. TS *P* = 0.0006). In the TS + SM group, only the abundance of the trimethoprim increased (NC vs. TS + SM *P* = 0.0004), while the others seem to be no changes. Notably, the abundance of the cephalosporin decreased in two groups (NC vs. TS *P* = 0.0001; NC vs. TS + SM *P* = 0.0002), but the change was most pronounced in the TS group. The above changes were also observed in the cluster heat map shown in Fig. 7c.

## Discussion

During the execution of space missions, astronauts are not only affected by changes in the space environment which mainly include weightlessness, high radiation, and hypoxia^17,39^, but are also restricted inside a narrow and closed cabin environment. Under these conditions, changes in the symbiotic intestinal flora of the intestines of astronauts may occur and further affect their physical health even further influence the success of a space mission. The development of complex molecular biology technology has allowed us to conduct different types of research studies^30^. In order to further explore the influence of complex space environments on the intestinal flora, we used the ground-based simulated module and a TS model to explore the influence of a combined effect on changes in species composition, function, and resistance genes of intestinal flora in mice.

In the present study, we found that different environments could cause changes in the species composition of intestinal flora in mice and these changes were distributed at different levels. This is consistent with the results of the species annotation of the intestinal flora of crewmembers in the ground-based space simulation study conducted by Turroni^40^. Especially at the phylum and genus levels, the effects of a simulated microgravity environment and combined effects environment on the mice can be observed on species composition. Specifically, when compared with the NC group, *Firmicutes* showed an increasing and decreasing trend in the TS group while *Bacteroidetes* decreased in the TS + SM group at the phylum level. Under normal circumstances, *Firmicutes* and *Bacteroidetes* are the main components of intestinal flora^41^. *Firmicutes* may metabolize carbohydrates to produce butyrate, which is beneficial not only for energy metabolism but also for intestinal mucosa protection and immune function^42^. *Firmicutes* is a class of gram-positive bacteria and one of the largest families in the domain bacteria. Most *Firmicutes* bacterial walls contain high levels of peptidoglycans (50%-80%) and are thick enough to form spores, resulting in them being highly resistant to dehydration or extreme environments^43^. Besides, several studies have shown that a large number of *Firmicutes* in the gut are associated with obesity^44–46^. Although obesity would have been a knock-out criterion so that it wouldn't have happened to astronauts, its associated metabolic changes require further attention due to its metabolic dysfunction. Several studies have demonstrated that many changes in the lipid metabolism of gastric cancer cells and epidermal stem cells have been observed under simulated microgravity^47, 48^. *Bacteroidetes*, a core flora of the human intestinal tract, contain a powerful polysaccharide degrading system to digest dietary fibers consumed by the body and convert them into short-chain fatty acids^49^. Relying on its powerful metabolic capacity, the dominant position of *Bacteroidetes* in the absorption of nutrients in the human body is unrivaled^50^. However, this role depends on the balance between nutrient absorption and consumption in the body^51^. Besides, the main source of Vitamin K in humans is also synthesized by *Bacteroides*^52^. Vitamin K deficiency affects various systems in the body, such as the coagulation system and the musculoskeletal system^53,54^. Due to these important functions, *Bacteroidetes* are widely regarded as beneficial bacteria that can decrease intestinal inflammation, immune dysfunction, and metabolic disorders, and may even function in preventing the occurrence of cancer^55^. Moreover, the metabolites of *Bacteroides*, such as propionate and acetate, can also block the absorption of enteric endotoxin^56^ and induce the apoptosis of colon cancer cells^57^, playing a preventive and therapeutic role. Kuhn et al. found that the specific mechanism by which the protective role of *Bacteroides* is exerted may be through recruitment of intraepithelial lymphocytes to produce IL-6^58^. Overall, *Bacteroides* is a protective barrier of the host intestinal tract^59^. However, in certain cases, *Bacteroidetes* can also become opportunistic pathogens that can cause diseases^60^. Although there was a decrease in the number of *Bacteroidetes* in the present study, these changes need to be addressed due they indicate changes in immune function in space extreme environment.

In addition, we screened biomarkers that could be used at the species level for the effects caused by simulated weightlessness or complex environmental factors. The increase of *Bacteroidetes* in the NC group could be used as biomarkers, which suggested that the relative abundance of *Bacteroidetes* decreased under weightless environment and combined effects environment. This was consistent with the results obtained through species annotation, and also indicated that the changes in *Bacteroidetes* abundance in combined effects environment were very important for health.

The KEGG enrichment analysis showed that compared with the NC group, the proportion of metabolic pathways under the two different environments decreased, while the decrease was most obvious in the combined effects environment. The changes in these metabolic pathways were highly correlated with changes in intestinal floral species composition. To respond to environmental changes, the functional genes involved in environmental information processing of the gut microbiota were increased in the combined effects environment, which is consistent with the Liu’s research on the change of intestinal flora function in astronauts^61^. Further studies are needed to elucidate the possible effects of these environmental change-induced functional changes on the body and intestinal flora. It is worth noting that changes in intestinal flora species composition and functional genes in mice were not consistent with that of weightlessness and combined effects environment, which needs to be confirmed through further studies.

The abuse of antibiotics leads to irreversible changes in the human body and microbial communities in the environment, which poses risks to human health and the ecological environment^62–64^. Therefore, the study of resistance genes has attracted extensive attention from researchers. The changes of abundance in resistance genes in intestinal flora may provide new insights for the application of antibiotics and the prevention of diseases. The weightless environment and combined effects environment both significantly affected the abundance of resistance genes in the intestinal flora of the mice. Although the total number of resistance genes was decreased in the TS and TS + SM groups, the fluctuation in the expression of the resistance genes was more meaningful. For example, the decreased abundance of the cephalosporin in two groups may provide guidance on medication for astronauts, which indicated that the treatment effect of cephalosporin still encouraging.

The manned spaceflight environment is a complex environment that is subjected to multiple physical changes. In this study, we focused on the effects of combined effects environment on species composition and function of intestinal flora in mice, but more studies on the effect of complex factors on intestinal flora are needed to confirm the applicability of these results. In addition, the specific reasons for the changes in intestinal floral species composition and functions in mice under weightlessness or complex factors need to be further confirmed. Based on previously reported results, it remains to be determined whether there is a correlation between the changes in immune function and the changes in intestinal flora microecology under a microgravity environment, while mutual influences induced by the two groups, as well as the recovery of intestinal flora after exposure to a space environment are also worthy of attention.

## Conclusion

We conducted this study to explore changes in intestinal flora of mice under the influence of different space environmental factors to enrich our understanding and provide a theoretical basis for space medical supervision and healthcare. The results showed that different environments, especially a combined effects environment could significantly alter the species composition and function of intestinal flora in mice. Besides, the changes in ARGs under the influence of these environmental factors may be useful for the treatment and prevention of diseases. These changes in intestinal flora caused by exposure to the space environment should be taken seriously to avoid the adverse effects induced by participating in space missions. More importantly, further studies are warranted to analyze the mutualistic relationship between humans and gut microbiota under complex environmental conditions.

## Methods

### Preparation of experimental animals and collection of specimens

Nine male C57BL/6 mice with the same genetic history were randomly divided into three groups: normal control (NC) group (normal environment), tail-suspension (TS) group (simulated microgravity environment), and tail-suspension in simulated module (TS + SM) group (the combined effects environment). The TS model was constructed using the Globus method and the mice were suspended by their tails to restrict movement^65^. The ground-based simulated module (SM) for animal research was used to build compound factors of the space environment. It contains 200 lx Light intensity, the circadian rhythm of 12 h light/darkness respectively; Atmosphere pressure (AP): 0.9 atm pressure; and 85 dBA environment noise. The experiment period for all three groups was 45 days. The level of AP was set according to data from China's spacecraft^66^. Noise level less than 100 dBA usually does not cause permanent hearing threshold shift (PTS) for mice^67^. During the first 7 days, all mice were allowed to settle under normal conditions (1 atmosphere and noiseless, 10 dbA) to adapt to the new environment. Beginning from the 8th day, mice were kept under different environmental conditions based on their experimental grouping. An ambient temperature of 25 degrees was maintained. In addition, because the SM was shielded and was not affected by external light, the two other groups of mice were artificially exposed to the same lighting conditions. The total duration of the experiment was set to 45 days to simulate a long-term spaceflight (usually > 1 month)^68^. At the end of the 45th day, the fecal samples were collected from the ileocecal region for further analysis. All animal experiments were approved by the Institutional Animal Care and Use Committee of the Strategic Support Force Medical Center.

### Sample preparation and treatment

The fecal samples (50 mg) were weighed in 1 ml microcentrifuge tubes and placed in liquid nitrogen and were subsequently stored at -80℃ until used. Total DNA was extracted from frozen fecal samples for metagenomic sequencing using a QIAamp Fast DNA stool Mini Kit (QIAGEN, Inc., Germany). The whole extraction process was performed as instructed by the manufacturer. Agar-gel electrophoresis (AGE), Nanodrop, and Qubit 3.0 system (Thermo Fisher Scientific, Inc., America) were used to determine the purity and integrity of the extracted DNA. DNA of sufficient purity and integrity were tested for library construction and sequenced using an Illumina HiSeq high-throughput sequencing platform (Qinglian Biotec. Co., Ltd, Beijing, China) along with a KAPA Hyper Prep Kit (KAPA Biosystems, Inc., America). The raw data obtained from the sequencing were further filtered to obtain a higher quality of clean reads for subsequent informational analysis. SOAPdenovo Assembly software was used to assemble the Clean Data and Scaftigs were obtained^69^. The scaftigs were further filtered for statistical analysis and subsequent genetic prediction (fragments cut-off: 500 bp).

### Data analysis

The Open Reading Frames (ORFs) were predicted and mapped using CD-HIT and SoapAligner software and were filtered using the scaftigs^70,71^. Redundant genes were eliminated to obtain the gene catalogue (Unigenes). The abundance information of each gene in each sample was calculated and visualized using a violin diagram. A Venn diagram was used to present the differently expressed genes. Then, DIAMOND software^72^ (Version 0.7.9; https://github.com/bbuchfink/diamond/) was used to blast and compare the Unigenes with sequences of the bacteria, fungi, archaea, and viruses extracted from the NR database (Version: 20161115; https://www.ncbi.nlm.nih.gov/) of NCBI (e-value < 0.05). An LCA algorithm was used for species annotation to obtain abundance information of each sample at various classification levels. PCA and NMDS were used to analyze the correlation between samples. All annotated results were visualized using Krona and heat maps. LEfSe multivariate statistical analysis was used to screen the biomarkers of the representative groups. Then, the putative amino acid sequences were translated from the gene catalog and aligned against the proteins/domains in the KEGG and eggNOG databases using default setting^73,74^. Subsequently, the Antibiotic Resistance Genes Database (ARGD) was used to annotate the Unigenes to antibiotic resistance genes (ARGs)^75^. The ARGs were classified and the specific antibiotics tolerated by them were also identified. Meanwhile, relative abundance analysis and abundance clustering analysis were performed based on the results of each sample.

### Ethics declarations

The authors are accountable for all aspects of the work in ensuring that questions related to the accuracy or integrity of any part of the work are appropriately investigated and resolved. Experiments were performed under a project license (NO.: K2019-098-01) granted by institutional ethics board of Strategic Support Force Medical Center, in compliance with China national or institutional guidelines for the care and use of animals. This study does not involve gene editing, nor does it involve bacteria or virus infection experiments, but mainly physiological and behavioral experiments, so there is no potential harm from these aspects. In the process of handling animals, we treated animals well according to the regulations of animal ethics and properly disposed of animal carcasses according to the regulations. And our experiments were performed in accordance with ARRIVE guidelines.

## Supplementary Information

Supplementary Tables.

## Data Availability

The datasets generated and/or analyzed during the current study are not publicly available due request of the project sponsor but are available from the corresponding author on reasonable request.

## Abbreviations

SCFAs: Short-chain fatty acids
ARGs: Antibiotic resistance genes
ORFs: The Open Reading Frames
Unigenes: The gene catalogue
AGE: Agar-gel electrophoresis
CARD: Comprehensive antibiotic resistance database
PCA: Principal component analysis
NMDS: Non-metric multidimensional scaling
LEfSe: Linear discriminant analysis Effect Size
LDA: Linear discriminant analysis
NC: Normal control
TS: Tail-suspension
SM: Simulated module
