# Supplementary material for: The effects of combined environmental factors on the intestinal flora of mice based on ground simulation experiments
Source: Sci Rep. 2021 May 31;11:11373. doi: 10.1038/s41598-021-91077-7 (PMC8166921; doi:10.1038/s41598-021-91077-7)
Supplement: Supplementary file 1 — Supplementary Tables. [file 41598_2021_91077_MOESM1_ESM.docx]

**Title page**

The effects of combined environmental factors on the intestinal flora of mice based on ground simulation experiments

Peiming Sun^1#^, Jiaqi Yang^1,2#^, Bo Wang^3^, Huan Ma^4^, Yin Zhang^4^, Jinhu Guo^4^, Xiaoping Chen^3^, Jianwei Zhao^3^, Hongwei Sun^1^, Jianwu Yang^1^, Heming Yang^1*^, Yan Cui^1*^

^1^ Department of General Surgery, Support Force Medical Center, Chaoyang District, Beijing 100101, China

^2^ Department of General Surgery, The 306th Hospital of Chinese PLA-Peking University Teaching Hospital, Chaoyang District, Beijing 100101, China

^3^ China Astronaut Research and Training Center, Haidian District, Beijing, 100094, China

^4^ Ministry of Education (MOE) Key Laboratory of Gene Function and Regulation, State Key Laboratory of Biocontrol, School of Life Sciences, Sun Yat-sen University, Haizhu District, Guangzhou, 510006, China

^#^ Author contributed equally to this work.

Corresponding authors: Yan Cui, email: dryancui@163.com; Heming Yang, email: yhming306@163.com.

Running title: Combined effects environment on the intestinal flora of mice

Figure and table: This manuscript contains a total of 7 figures and 3 tables.

**Tables**

**Table S1.** The processing information of the Raw Data

| **Sample**  **ID** | **Raw Data**  **(Mb)** | **Clean Data**  **(Mb)** | **Clean Q20**  **(%)** | **Clean Q30**  **(%)** | **Clean GC**  **(%)** | **Effective**  **(%)** |
| --- | --- | --- | --- | --- | --- | --- |
| NC-1 | 12920.6 | 9779.23 | 100 | 99 | 49.79 | 75.69 |
| NC-2 | 12379.45 | 9505.48 | 100 | 99.01 | 48.81 | 76.78 |
| NC-3 | 13517.56 | 10386.27 | 100 | 99.02 | 48.9 | 76.84 |
| TS-1 | 13275.95 | 10353.39 | 100 | 99.07 | 48.78 | 77.99 |
| TS-2 | 14259.42 | 11370.98 | 100 | 99.09 | 49.46 | 79.74 |
| TS-3 | 13395.55 | 10458.76 | 100 | 99.12 | 47.86 | 78.08 |
| TS+SM-1 | 11083.65 | 8170.84 | 100 | 98.99 | 46.28 | 73.72 |
| TS+SM-2 | 13787.34 | 10604.47 | 100 | 99.04 | 46.38 | 76.91 |
| TS+SM-3 | 15457.35 | 12321.5 | 100 | 99.13 | 45.67 | 79.71 |

**Table S2.** The assembly results of the samples

| **Sample**  **ID** | **Total len**  **(bp)** | **Contigs**  **Number** | **Average len**  **(bp)** | **N50 len**  **(bp)** | **N90 len**  **(bp)** | **Max len**  **(bp)** | **Min len**  **(bp)** |
| --- | --- | --- | --- | --- | --- | --- | --- |
| NC-1 | 147408755 | 59335 | 2484.35 | 8613 | 753 | 190288 | 500 |
| NC-2 | 142996396 | 60435 | 2366.12 | 7634 | 730 | 206425 | 500 |
| NC-3 | 138450063 | 63855 | 2168.19 | 6251 | 699 | 269223 | 500 |
| TS-1 | 329782389 | 196045 | 1682.18 | 2664 | 639 | 201216 | 500 |
| TS-2 | 368818073 | 209508 | 1760.4 | 2803 | 664 | 226738 | 500 |
| TS-3 | 304792667 | 172639 | 1765.49 | 3095 | 645 | 232085 | 500 |
| TS+SM-1 | 125159110 | 67500 | 1854.21 | 4274 | 612 | 173432 | 500 |
| TS+SM-2 | 152759811 | 93519 | 1633.46 | 4079 | 572 | 177716 | 500 |
| TS+SM-3 | 192480689 | 151212 | 1272.92 | 1927 | 545 | 197067 | 500 |

**Table S3.** Basic Information of the Gene Catalogue

| **ORFs NO.** | 939,480 |
| --- | --- |
| **integrity:all** | 405,674(43.18%) |
| **integrity:start** | 207,006(22.03%) |
| **integrity:end** | 209,080(22.25%) |
| **integrity:none** | 117,720(12.53%) |
| **Total len.**  **(Mbp)** | 617.65 |
| **Average len.**  **(Mbp)** | 657.43 |
| **GC percent** | 50.28% |
